# Teleworking in Times of COVID-19: Effects on the Acquisition of Personal Resources

**DOI:** 10.3389/fpsyg.2021.685275

**Published:** 2021-06-23

**Authors:** Manuel Pulido-Martos, Daniel Cortés-Denia, Esther Lopez-Zafra

**Affiliations:** Departamento de Psicología, Psicología Social, Universidad de Jaén, Jaén, Spain

**Keywords:** COVID-19, labor resources, social support, telework, vigor, well-being at work

## Abstract

The COVID-19 pandemic has forced many employees to introduce different degrees of teleworking, leading to a transformation of the psychosocial work environment. In this study, we analyzed whether the relationship between a labor resource, social support, and a personal resource, vigor at work, is affected by the work modality (face-to-face, hybrid that includes face-to-face work and telework time, and telework caused by the current pandemic situation). Five hundred and forty-three employees answered an online questionnaire about their perceptions of the levels of social support, vigor experienced in the last month, and work modality. Seniority in the organization and the gender of the employees were controlled for. The model fit was significant [*F*_(7, 535)_ = 20.816, *p* < 0.001], accounting for 21% of the variation in vigor (*R*^2^ = 0.21). The interaction was also significant [*F*_(2, 535)_ = 4.13, *p* < 0.05], with an increase of 1% in the explanation of the variance in vigor at work (Δ*R*^2^ = 0.01). Differences were found in the positive relationship between levels of social support and vigor at work, among the face-to-face and telework modalities (hybrid and telework), but not between teleworking modalities. As a result, we posit that the different forms of telework moderate (buffer) the relationship experienced between labor resources (social support) and personal resources (vigor at work). This implies that, for the design of teleworking conditions, it is necessary to provide work resources similar to those in face-to-face settings, such as social support.

## Introduction

The health crisis caused by the COVID-19 (an infectious disease caused by the SARS-CoV-2 virus) pandemic, declared by the World Health Organization on 11 March 2020 (World Health Organization, 2020), along with the lockdown of large populations, forced numerous organizations to establish teleworking to ensure the health and safety of workers and the maintenance of economic activity (Belzunegui-Eraso and Erro-Garcés, 2020; Bouziri et al., 2020; Morilla-Luchena et al., 2021). In this sense, the pandemic has extensively generated new forms of work, introducing different degrees of telework (complete telework or hybrid, a part of telework and another part of face-to-face work). In this line, Allen et al. (2015, p. 44) defined telework as “a work practice that involves members of an organization substituting a portion of their typical work hours (ranging from a few hours per week to nearly full-time) to work away from a central workplace – typically from home – using technology to interact with others as needed to conduct work tasks.” Although this new formula leads to greater health protection by slowing the expansion of the COVID-19 among workers by increasing the social and physical distances, this social isolation could also impact the mental health of employees (Lengen et al., 2021). Thus, the relationships between this new way of working, employee performance, and health are complex (Vander Elst et al., 2020) and should be further analyzed.

Following the theory of job demands-resources (JD-R; Demerouti et al., 2001; Schaufeli and Bakker, 2004), in this study, various resources are considered. This theory posits that the work demands (i.e., physical, psychological, social, and organizational aspects involving sustained physical and/or psychological efforts and associated with physical and/or physiological costs) can be an obstacle when they require a lot of effort, and the resources (i.e., physical, psychological, social, and organizational aspects that enable goals to be achieved at work) reduce work demands and associated psychological and physiological costs or stimulate personal growth, development, and learning, making it possible to meet the job demands (Bakker and Demerouti, 2007; Bakker, 2011). Thus, work is likely to require an effort that consumes energy resources, whereas other resources would cushion the impact of these demands. In this vein, telework seems to have a considerable impact on the quality and quantity of labor demands and resources. Specifically, in relation to demands, telework increases overload, interruptions, misunderstandings, and conflicts and decreases emotional work. In terms of resources, telework increases feedback and autonomy and decreases career advancement and social support (Demerouti et al., 2014). However, it does not always have to produce a reduction in social support, for example, Collins et al. (2016) posited that working from home can also allow teleworkers to seek and develop greater social and labor support relationships with other teleworkers, permitting them to get emotional support about a work situation or just to catch up on personal issues. Moreover, technology can also maintain the social interaction with peers out of working time and space (Lal and Dwivedi, 2009). Therefore, it could be considered that the mode of teleworking could have a moderating role (Duxbury and Halinski, 2014) between social support and other personal resources (Othman and Nasurdin, 2013; Yu et al., 2019). Could it be the same in a pandemic situation?

The figures concerning telework prior to the COVID-19 crisis indicated that, in 2018, only 4.3% of Spanish workers and 5.2% of European Union (EU) workers worked at home (Eurostats, 2020), whereas during the COVID-19 crisis, early estimates suggest a much larger prevalence than before the crisis. For example, Eurofound (2020) estimated that close to 40% of those currently working in the EU began to telework full time as a result of the pandemic. Thus, the abrupt and unplanned incorporation of these new working modalities, due to the pandemic, has led to a transformation of the psychosocial environment at work, altering different labor and personal resources in workers. In this sense, Salanova et al. (2010) showed that both types of resources influence each other by creating a “positive profit spiral.” These cycles of feedback between labor and personal resources can be explained through theories such as the conservation of resources (COR; Hobfoll, 1989, 2002). This theory poses that people strive to protect, preserve, and increase their resources and that, in addition, resources are not held in isolation but tend to be clustered, allowing the possession of certain resources to lead to the procurement of additional resources (Hobfoll et al., 2018). Thus, the threat or loss of one or more resources would encourage to protect them, but not to create or acquire new resources, whereas those workers who obtain new or maintain resources are prone to the creation of new resources, thereby generating a positive profit spiral. The personal resources included in the JD-R theory are cognitive and related to a resilience function (Xanthopoulou et al., 2009; Airila et al., 2014). However, resources of a social nature, or even related to personal physical energy, have not been considered, although they tend to relate to each other, which could reveal the existence of a common nucleus (Mayerl et al., 2016). A fundamental social resource is social support at work, defined as the social interaction available at the workplace involving relationships with coworkers and supervisors (Karasek and Theorell, 1990). This support can provide emotional support, related to listening and comforting colleagues; instrumental support which is more tangible and related to the provision of materials and services needed to perform the work; and information support, which would provide information and advice (Sias, 2009). Bearing in mind, the interplay between resources, social support at work, such as a work resource, could influence other types of resources, such as personal resources. If we consider the recent revisions of the COR theory (Halbesleben et al., 2014), whatever a person perceives as helping them reach a goal or objective would be considered a resource; thus, the vigor at work (Shirom, 2004) could be a personal resource.

Vigor at work is a positive affection composed of three dimensions: physical strength, emotional energy, and cognitive liveliness (Shirom, 2004, 2011). Physical strength has to do with the physical abilities of a person. In contrast, emotional energy refers to the ability to express empathy and a positive orientation toward establishing relationships with other coworkers. Cognitive liveliness is related to mental agility and the ability to contribute new ideas. Thus, feeling vigor at work would imply a feeling of moderate activation that is accompanied by an experience of pleasure. In this way, vigor could be considered a complete personal resource that not only includes cognitive aspects but also addresses the social and physical dimensions of a person. In addition, it would be a result of work experience and, as with other positive affects, would facilitate target-oriented behavior (Carver and Scheier, 1990) and approximate behavior (Watson, 2000). This makes it *de facto* a personal resource resulting from work experiences (Halbesleben et al., 2014).

This study, based on the JD-R and COR theories, aimed to analyze the implications of the relationship between a labor resource experienced at work, such as the social support of colleagues and supervisors, and a personal resource, such as the vigor at work. Moreover, we consider the modality of work to compare the face-to-face work with the hybrid modality (face-to-face work and telework) and telework. In the latter case, to avoid bias, workers had to begin teleworking due to the COVID-19 pandemic. Specifically, we propose:

*Hypothesis* 1: Social support will be positively related to the levels of vigor at work.
*Hypothesis* 2: The modalities that perform some type of telework decrease the slope of the regression line, between the relation of social support and vigor at work, compared to the face-to-face type of work.

## Materials and Methods

### Design, Participants, and Procedure

A cross-sectional online survey was conducted with self-report measures of workers from different sectors of activity. From the initial 594 participants, 51 were eliminated for not paying enough attention to the task. Following the procedure of Maniaci and Rogge (2014), we included an item that asked participants to answer with a specified response (e.g., “If you are reading this question answer with 2”) to detect inattention. Thus, the final sample was composed of 543 workers (55.2% women) with a mean age of 38.6 years (*SD* = 11.7; range 19–63 years) and a mean seniority at the organization of 10.08 years (*SD* = 9.99; range 0.5–41 years). In terms of work modality, 37.2% were face-to-face workers, 30.4% were teleworkers, and 32.4% had a hybrid modality.

The workers participated voluntarily and were recruited through students from different locations, earning psychology, social work, and social education degrees at the University of Jaén (Spain). The data were collected from October 2020 to January 2021. The students were instructed on the procedure and distribution of the survey, following the protocol approved by the Ethics Committee of the University of Jaén (Ref. NOV.19/1.PROY).

### Measures

#### Sociodemographics

Workers reported their gender, age, seniority in the organization, and working modality, i.e., face-to-face, hybrid mode, or telework. In the last case, they reported whether they began this modality due to COVID-19. Only the workers answering “yes” to this question were considered.

#### Social Support Received by Peers and Superiors at Work

*Social support* was measured through the social support dimension of the Job Content Questionnaire (JCQ; Karasek and Theorell, 1990; Spanish adaptation by Escribà-Agüir et al., 2001). This dimension comprises nine items (e.g., “The people I work with are interested in me”) with a four-point Likert response format ranging from 1 (totally disagreeing) to 4 (totally agree). The internal consistency of this dimension was adequate (*α* = 0.91).

#### Vigor

The Shirom-Melamed Vigor Measure (SMVM; Shirom, 2004; Spanish adaptation by Pulido-Martos et al., 2019) was used to measure vigor at work. The scale consisted of 12 items that comprised the following three dimensions: physical strength (five items; e.g., “I feel full of energy”), cognitive liveliness (three items; e.g., “I feel I can contribute with new ideas”), and emotional energy (four items; e.g., “I feel able to show warmth to others”). The response format ranged from 1 (almost never) to 7 (almost always). The scale yielded adequate reliability (*α* = 0.92).

## Results

### Data Analysis

IBM SPSS was used for descriptive statistics, Pearson’s correlation coefficients, and intergroup ANOVAs. The macro PROCESS (Hayes, 2018), from SPSS, was used for moderation analysis.

### Descriptive Statistics

Descriptive statistics and Pearson’s correlation coefficients derived from the analyses, including those for control variables, are shown in Table 1. The levels of social support and vigor at work experienced by employees were positively and significantly related (*r* = 0.40, *p* < 0.01). Years of seniority in the organization and the gender of participants were not significantly related to any of the variables in the study. However, both were controlled for the regression analysis.

**Table 1:** Pearson correlations and descriptive statistics for all variables.

	Variable	1	2	3	4
1	Seniority at organization	-	−0.028	−0.075	−0.039
2	Gender		-	−0.002	0.067
3	Social support			-	0.402^∗∗^
4	Vigor				-
Descriptive statistics	Mean (*n*)	10.08	300	3.25	5.62
	*SD* (%)	9.99	55.2	0.62	0.88

Seniority at organization, years as a worker in the organization; Gender, man = 0, woman = 1; and descriptive statistics for column 2 refer to the number and percentage of female workers.

∗∗ *p* < 0.01.

### Differences Among Work Modalities

Table 2 shows the univariate unifactorial between-group ANOVA results. Statistically significant differences were found between the groups according to the mode of work (face-to-face, hybrid, and teleworking) and the levels of vigor in the work experienced by the participants [*F*_(2, 540)_ = 5.362, *p* < 0.01, *η*^2^ = 0.02]. Tukey’s test which was used to analyze differences in the mean levels of vigor between the groups revealed that employees with teleworking modality (*M* = 5.44; *SD* = 0.89) showed lower levels of vigor than those with the face-to-face (*M* = 5.72; *SD* = 0.93) and hybrid (*M* = 5.68; *SD* = 0.81) modalities. No significant differences were found in vigor at work between the hybrid and face-to-face modalities. Regarding possible differences in the level of perceived social support, although the analysis indicated that the groups were not the same [*F*_(2, 540)_ = 3.065, *p* < 0.05, *η*^2^ = 0.01], pairwise comparisons of the means using the Tukey’s test did not reveal significant differences, possibly due to the more conservative nature of this test (Howell, 2010).

**Table 2:** Vigor and social support by work modality.

	Face-to-face		Hybrid		Telework				
	*M*	*SD*	*M*	*SD*	*M*	*SD*	*F*_(2, 540)_	*p*	Contrasts
Vigor	5.72	0.93	5.68	0.81	5.44	0.89	5.362	0.005	a>c;b>c
Social support	3.21	0.63	3.21	0.67	3.35	0.56	3.065	0.047	-

a, Face-to-face; b, Hybrid; and c, Telework.

### Moderation Analyses

**Figure 1:**
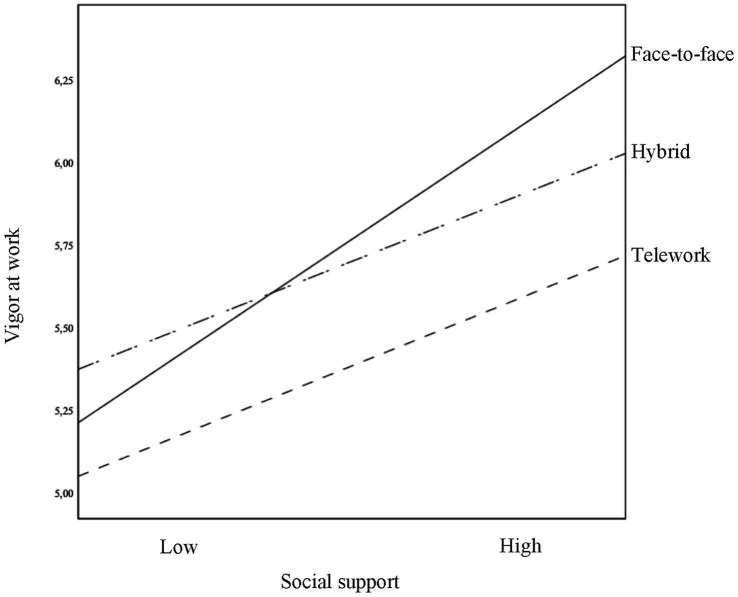
Regression lines showing the effects of the interaction between work modality and social support in the prediction of levels of vigor at work. High and low social support values represent 1 SD above and below the average of the sample.

A moderation model was used to test whether the relationship between the perceived levels of social support and that of vigor at work depended on the work modality. Using the macro PROCESS for SPSS (Hayes, 2018), the fit to the data of Model 1 was tested, taking the levels of vigor at work as a variable criterion, the social support perceived as the predictive variable, and the work modality (0 = face-to-face, 1 = hybrid, and 2 = telework) as the potential moderating variable. As work modality was a categorical variable, with three categories (*k* = 3), PROCESS generated *k*-1 dummy variables. The indicator coding option was chosen for the generation of these variables (Hayes and Montoya, 2017). Similarly, two interaction terms were introduced into the model to represent the possible interaction between the work modality and the levels of social support of employees. The possible effects of seniority on organization and gender were monitored. The general model results are displayed in Table 3. The fit of the model was significant [*F*_(7, 535)_ = 20.826, *p* < 0.001], accounting for 21% of the variance in vigor (*R*^2^ = 0.21). The control variables (gender and seniority) were not related to the levels of vigor. Social support levels showed a significant and positive relation with vigor at work (*β* = 0.79, *t* = 8.95, *p* < 0.001). The mode of work, represented by the terms D1 (*β* = −0.07, *t* = −0.81, *p* = 0.42) and D2 (*β* = −0.38, *t* = −4.58, *p* < 0.001), showed a negative and significant relationship only for D2. The interaction effects, represented by Int 1 (*β* = −0.32, *t* = −2.60, *p* < 0.01) and Int 2 (*β* = −0.32, *t* = −2.27, *p* < 0.05), were significant. An omnibus test of interaction effects testing was significant [*F*_(2, 535)_ = 4.15, *p* < 0.05], with the interaction effects explaining an additional 1% of variance in the level of vigor at work (Δ*R*^2^ = 0.01). Figure 1 allows us to interpret the interaction effects included in the model, which are shown by the differences between the slopes of the lines. From the levels of significance associated with the coefficients of the interaction terms, which are listed in Table 3, it is possible to affirm that the slopes for the two working modalities that include some form of teleworking are significantly different from those for the face-to-face modality. Following Hayes and Montoya (2017), we reran the regression analysis by changing the reference group to be able to check the differences in the slope of the lines between the hybrid modality and teleworking. No significant differences were found (*β* = −0.07, *t* = −0.81, *p* = 0.42).

**Table 3:** Regression analysis testing the effects of the interaction between levels of social support and the modality of work in the explanation of levels of vigor.

DV = Vigor	*β*	*SE*	*t*	*p*
Seniority	−0.00	0.00	−0.82	0.41
Gender	0.12	0.07	1.74	0.08
Social support	0.79	0.09	8.96	0.00
Mode D1	−0.07	0.08	−0.81	0.42
Mode D2	−0.38	0.08	−4.58	0.00
Int1: D1 × social support	−0.32	0.12	−2.60	0.00
Int2: D2 × social support	−0.32	0.14	−2.27	0.02

Working mode: 0 = face-to-face, 1 = hybrid, and 2 = telework; Codes: Mode D1: face-to-face = 0, hybrid = 1, and telework = 0; Code Mode D2: face-to-face = 0, hybrid = 0, and telework = 1; Int1 = D1 × social support; Int2 = D2 × social support.

## Discussion

Due to the importance of social support, especially in the lockdown situation due to the COVID-19 pandemic, this study aimed to analyze the implications of experienced work resources and personal resources (vigor at work), testing how they are affected according to the modality of work and comparing the face-to-face modality (37.2% of the workers) with the hybrid (32.4%) and telework modalities (30.4%), the latter of which is caused by the current pandemic situation.

Overall, the study hypotheses were confirmed; the results showed that social support, both from peers and supervisors, is positively and significantly related to the level of vigor at work (H1). Therefore, perceived social support would be a source of resilience to unavoidable challenges (Layous and Nelson-Coffey, 2020), such as the pandemic, confinement, and telework, increasing the level of vigor. Vigor at work has been shown to have important implications for the physical health of workers (Cortés-Denia et al., 2021). Thus, based on the resources of the JD-R theory, it was possible to verify how a labor resource influences a personal resource, confirming the feedback between these resources through the COR theory (Hobfoll, 1989, 2002). However, the relationship between these two resources is moderated, in the pandemic period, by the modality of work, buffering that relationship. Specifically, the relationship between social support and vigor at work is lower when teleworking (H2). Although some studies have found several advantages associated with telework in relation to greater flexibility of time and work–life balance (Madsen, 2003; Gajendran and Harrison, 2007), showing that technologies may maintain the social interaction among colleagues outside the working time (Lal and Dwivedi, 2009), this relation probably does not have the same quality and closeness, even if they perceive the same level of social support. This fact makes it possible for the relationship between social support and vigor at work to be conditioned by the modality of telework. In this line, the modality of teleworking could produce a decline in the quality of professional relations (Vayre and Pignault, 2014) and, at the same time, a decrease in experimenting the vigor at work, by a reduction in the effect on feeling physically and cognitively active. Probably, telework is likely to involve high demands, including overtime, given the continued availability of employees (Abendroth and Reimann, 2018), lowering energy levels of workers. Thus, considering COR theory (Hobfoll, 1989, 2002), it has been found that this feedback between a labor resource, such as peer support, and other personal resources, such as vigor at work, may be affected by changing working conditions, such as the shift from face-to-face to full or hybrid telework, generated by the pandemic, affecting this feedback of resources.

Regarding the practical implications of this study, probably the abrupt and unexpected introduction of the various forms of teleworking for the protection of the health of workers and the maintenance of economic activity, due to the COVID-19, has given priority to the design of teleworking, performance, and several aspects related to results, whereas other social resources have been neglected. For that reason, when promoting teleworking conditions, it is necessary to try not only to reproduce a working environment in which the demands are similar to those of the face-to-face model but also to provide similar labor resources, such as social support. Furthermore, other aspects related to the workplace such as the conciliation possibilities should be considered. The schedule in telework conditions used to be similar to the normal one in the workplace, but without considering the difficulties at home. Thus, the conciliation work–family can be affected. As a result, innovations in teleworking, for example, “smart working,” which have no specific restrictions on working hours or the workplace (Di Nicola, 2017), could provide greater flexibility and better conciliation between family and work.

However, this study also has some shortcomings. As a cross-sectional study, this study cannot indicate how the evolution of the situation affects workers; perhaps during the pandemic, organizational changes in the modality could also produce changes in how the situation is faced. Furthermore, we considered only teleworkers due to COVID-19, but not all jobs can be done at home (Dingel and Neiman, 2020), which may produce differences. Moreover, the prevalence of telework is different depending on the productive sector and even across countries (Milasi et al., 2020). Thus, a future study should consider comparisons between and within sectors along with comparisons of telework before and during the pandemic. Moreover, if the organization does not take care of the conclusions raised in this study related to telework, it would be interesting to analyze the relationship between social support and levels of vigor, depending on the teleworking conditions (particularly in times of pandemic), considering the use of personal initiatives, such as job-crafting (Gemmano et al., 2020; Ingusci et al., 2021), as a complementary way for obtaining resources, analyzing whether they have positive implications.

## Data Availability Statement

The raw data supporting the conclusions of this article will be made available by the authors, without undue reservation.

## Ethics Statement

The studies involving human participants were reviewed and approved by the Ethics Committee of the University of Jaén. The patients/participants provided their written informed consent to participate in this study.

## Author Contributions

MP-M, DC-D, and EL-Z conceived and designed the study and drafted the manuscript. DC-D and EL-Z trained the surveyors and collected the data. MP-M performed the measurements. MP-M and DC-D processed the data, performed the analyses, interpreted the data, and helped with the references. All authors critically revised the manuscript, approved this version, and agreed to be accountable for all aspects of this research and its integrity.

### Conflict of Interest

The authors declare that the research was conducted in the absence of any commercial or financial relationships that could be construed as a potential conflict of interest.
